# The Substituent Effect on the Radical Scavenging Activity of Apigenin

**DOI:** 10.3390/molecules23081989

**Published:** 2018-08-10

**Authors:** Yan-Zhen Zheng, Da-Fu Chen, Geng Deng, Rui Guo

**Affiliations:** 1 College of Bee Science, Fujian Agriculture and Forestry University, Fuzhou 350002, China; yanzhenzheng@fafu.edu.cn (Y.-Z.Z.); rui_0508@163.com (R.G.); 2 Key Laboratory of Bioorganic Phosphorous Chemistry and Chemical Biology (Ministry of Education), Department of Chemistry, Tsinghua University, Beijing 100084, China; dengg13@mails.tsinghua.edu.cn

**Keywords:** apigenin, substituent effect, structure–antioxidant activity relationship, Hammett sigma constants, density functional theory

## Abstract

Flavonoids widely found in natural foods are excellent free radical scavengers. The relationship between the substituent and antioxidative activity of flavonoids has not yet been completely elucidated. In this work, the antioxidative activity of apigenin derivatives with different substituents at the C3 position was determined by density functional theory (DFT) calculations. The bond dissociation enthalpy (BDE), ionization potential (IP), and proton affinity (PA) were calculated. Donator acceptor map (DAM) analysis illustrated that the studied compounds are worse electron acceptors than F and also are not better electron donors than Na. The strongest antioxidative group of apigenin derivatives was the same as apigenin. Excellent correlations were found between the BDE/IP/PA and Hammett sigma constants. Therefore, Hammett sigma constants can be used to predict the antioxidative activity of substituted apigenin and to design new antioxidants based on flavonoids. In non-polar phases, the antioxidative activity of apigenin was increased by the electron-withdrawing groups, while it was reduced by the electron-donating groups. Contrary results occurred in the polar phase. The electronic effect of the substituents on BDE(4′-OH), BDE(5-OH), PA(4′-OH), and IP is mainly controlled by the resonance effect, while that on BDE(7-OH), PA(5-OH), and PA(7-OH) is governed by the field/inductive effect.

## 1. Introduction

Free radicals in the body are natural by-products of metabolism. They are continuously generated by the use of oxygen like respiration and some cell-mediated immune functions. Free radicals have a key role in the initiation and advance of some serious diseases such as senescence, diabetes, cancer, and heart conditions [1]. In recent years, there has been growing interest in selecting efficient antioxidants to reduce the damage of free radicals.

**Figure 1:**
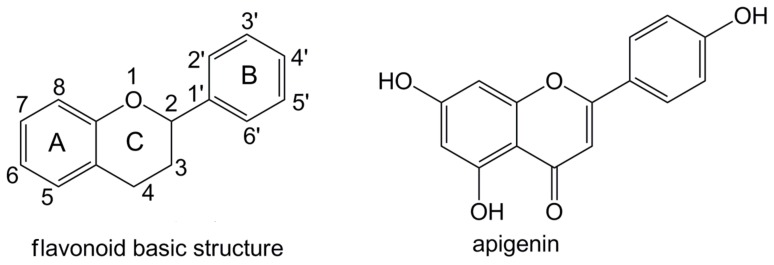
The flavonoid basic structure and the chemical structure of apigenin.

It is widely accepted that flavonoids found in various natural foods such as vegetables, beans, fruits, and bee products are excellent antioxidants [2]. Flavonoids are a class of plant secondary metabolites [2]. They consist of a benzene A ring condensed with an oxygenated heterocyclic C ring and phenyl B ring attached at C2 (Figure 1). According to their chemical structures, flavonoids are often subdivided into flavonols, flavanones, flavones, flavanols (catechins), anthocyanidins, and isoflavones. Due to the polyphenolic structure, flavonoids may scavenge various free radicals such as hydroxyl, superoxide, peroxyl, and lipid peroxyl radicals [3,4]. Acting as antioxidants, flavonoids exhibit some health benefits such as antiallergic, anti-inflammatory, antiviral, and anticancer activity [5,6,7].

One of the most abundant and most studied flavonoids is 4′,5,7-trihydroxyflavone, commonly known as apigenin (Figure 1). Apigenin is found in significant quantities in a variety of natural foods such as parsley, chamomile, oranges, celery, thyme, onions, honey, and bee pollen [8,9]. Interest in apigenin has grown in recent years due to its low intrinsic toxicity and remarkable effects on cancerous cells [10,11]. It is increasingly recognized that apigenin can be a chemopreventive agent of cancer [8,9,11]. It has been reported that apigenin can promote adverse metabolic reactions in vivo when consumed as part of a normal diet [10]. These beneficial effects could be largely attributed to its potent antioxidative activities [11].

In the past decades, many computational methods have been devoted to elucidate the antioxidative activity of phenolic compounds. They are able to provide reliable results in this research field with minimal costs. They are useful, especially when the experimental determination of the required properties or quantities is complicated. Computational methods, especially the DFT calculations, have been successfully used to evaluate the antioxidative activity of phenolic compounds [12,13,14,15,16,17,18,19,20,21,22,23]. Multiple DFT results have illustrated the close relationship between the antioxidative activity and structural property of phenolic compounds [12,13,14,15,16,17,18,19,20,21,22,23]. It seems that the antioxidative activity of flavonoid is mainly due to the molecular structures, more accurately to the number and character of the hydroxyl groups, the resonance effect, and the conjugation effect [17,19,23]. However, only the basic structures have been studied and the relationship between the substituent and their antioxidative reactivity has not yet been completely elucidated. Therefore, the aim of this study was to understand the structure–activity relationship of flavonoid derivatives with different substituents in radical scavenging reactions.

To understand the substituent effect on the antioxidative activity of flavonoids, DFT calculations were chosen as the tool and a series of apigenin derivatives with different groups (H, NH_2_, OMe, Me, OH, F, Cl, CHO, CF_3_, CN, and NO_2_) at the C3 position were selected for the detailed investigation. The substituents adequately contained the electron-donating groups and electron-withdrawing groups, and enabled informative comparisons from the results of the DFT calculations. The frontier orbital and donator acceptor map (DAM) of apigenin and apigenin derivatives were analyzed. Three main mechanisms: hydrogen atom transfer (HAT), single electron transfer followed by proton transfer (SET-PT), and sequential proton loss electron transfer (SPLET) by which antioxidants can undertake their important protective roles have been proposed [24,25,26]. The radical scavenging activities of the apigenin derivatives were studied based on these mechanisms, and then were compared with substituent sensitivity for each position, which were obtained from the correlation of Hammett sigma constants and BDE/IP/PA. Furthermore, the correlations between the electronic effects (field/inductive effect and resonance effect) and BDE/IP/PA were also considered to determine which effect played the dominant role. Apart from the gas phase, the benzene and water phases were used as models to simulate environments (physiological lipids and fluids) found in many foods and body tissues.

## 2. Results

### 2.1. Frontier Orbitals Analysis

The distribution and energy of the frontier orbital are important molecular parameters correlated with antioxidative activity. Figure S1 (in the Supplementary Materials) show the calculated distributions and energies of the frontier orbitals for the studied flavonoids in the gas phase. It can be seen that the HOMO orbitals and LUMO orbitals of the investigated compounds presented similar distributions with each other. More specifically, the HOMO orbitals were mainly localized on the A ring, B ring, and the region around the C2-C3-C4, whereas the LUMO orbitals were delocalized over the whole molecule.

The energy of the HOMO orbital is related to the electron-donating ability of a flavonoid. The higher the HOMO orbital energy, the stronger electron-donating ability of the compound [15,21,22]. In Figure S1, it can be observed that the electron-withdrawing groups such as CHO, CF_3_, and CN reduced the HOMO orbital energy and the electron-donating groups such as NH_2_, Me, and OMe raised the HOMO orbital energy. Thus, the electron-donating capacity would be strengthened by the electron-donating groups at the C3 position and was strongest for the NH_2_-substituted derivative.

### 2.2. Donator Acceptor Map Analysis

DAM is a useful tool for classifying any compounds in terms of its electron donating–accepting capacity [27,28,29]. In this work, F and Na atoms were selected as the reference to study the antioxidative activity of apigenin and its derivatives. A plot of electron donation index (*R*_d_) vs. electron acceptance index (*R*_a_) providing the DAM for apigenin and its derivatives in the gas phase is shown in Figure S2 (in the Supplementary Materials). The inset is definition of the four regions in DAM. Using the DAM, a compound can be classified in terms of its electron donating–accepting capability respected to F and Na. All of the *R*_a_ is less than 1, thus, the studied compounds are worse electron acceptors than F. All of the *R*_d_ is larger than 1, indicating that apigenin and its derivatives are not better electron donors than Na.

### 2.3. Bond Dissociation Enthalpy, Ionization Potential, and Proton Affinity Analysis

HAT is a one-step mechanism and the antioxidative activity of the investigated compound was determined by BDE in this mechanism. SET-PT and SPLET mechanisms consist of two steps. The first step is thermodynamically significant and determines the antioxidative activity of the flavonoid. Therefore, the following analysis mainly focused on the BDE of the HAT mechanism, IP of the SET-PT mechanism, and PA of the SPLET mechanism. Hammett sigma constants are derived from dissociation constant values of substituted benzoic acids in water. They reflect the strengths of the benzoic acids, which are related to the electronic withdrawing or donating capabilities of substituents attached to the aromatic moiety [30]. They are also one of the most widely used parameters to study and interpret the organic reactions and their mechanisms [31,32,33]. In this work, the Hammett sigma constants: *σ*_m_ and *σ*_p_ of the substituent were used. The electronic effect of the substituent is composed of two main parts: the field/inductive effect represented by parameter *F*, and the resonance effect characterized by parameter *R* [31,32,33]. The Hammett sigma constants (*σ*_m_ and *σ*_p_), field/inductive parameter (*F*), and resonance parameter (*R*) of the substituents, which were collected from the present work [31].

#### 2.3.1. Bond Dissociation Enthalpy

The computed BDEs of 4′-OH, 5-OH, and 7-OH in the investigated compounds are listed in Table 1. As can be observed in Table 1, the lowest BDEs were at 4′-OH for the investigated compounds in the studied media. Therefore, the 4′-OH of the apigenin derivatives underwent the HAT mechanism with the most possibility. The strongest antioxidative group of the apigenin derivative was the same as apigenin in different phases. The lowest BDE of NH_2_-substituted derivative was smaller than that of the other compounds. Thus, in the HAT mechanism, the NH_2_-substituted derivative was the strongest antioxidant among the studied compounds.

**Figure 2:**
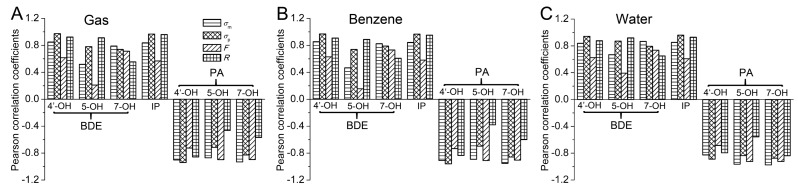
The Pearson correlation coefficients between the Hammett sigma constants (*σ*_m_ and *σ*_p_)/the field/inductive parameter (*F*)/resonance parameter (*R*) and the BDE/IP/PA in the (**A**) gas, (**B**) benzene, and (**C**) water phases.

The Pearson correlation coefficients between the Hammett sigma constants/the field/inductive parameter/the resonance parameter and the BDEs are drawn as histograms in Figure 2. As can be seen in Figure 2, all of the *P* (BDE, σ) were positive and larger than 0.8, indicating that the BDEs were positively and highly relevant with the Hammett sigma constants. The positive coefficient also suggested that the electron-withdrawing groups raised the BDE of the O-H groups in apigenin and the electron-donating groups had the opposite effect. Therefore, the electron-withdrawing groups at the C3 position reduced the antioxidative activity of apigenin while the electron-donating groups had the opposite effect.

For 4′-OH and 5-OH, the *P* (BDE, *σ*_p_) were larger than *P* (BDE, *σ*_m_) and exceeded 0.9 while for 7-OH, the *P* (BDE, *σ*_m_) were larger than *P* (BDE, *σ*_p_) and more than 0.9. Therefore, the correlation between the BDE(4′-OH)/BDE(5-OH) and *σ*_p_ was better than that between the BDE(4′-OH)/BDE(5-OH) and *σ*_m_ while the opposite result occurred for 7-OH. Furthermore, the BDE of 4′-OH and 5-OH in the substituted apigenin could be predicted better by *σ*_p_, while that of 7-OH could be forecasted well by *σ*_m_. The linear formulas related with the Hammett sigma constants and the BDEs are listed in Table S1 (in Supplementary Materials), which can be used to predict the BDEs by the Hammett sigma constants.

In Figure 2, it can also be observed that the correlation between BDE(4′-OH)/BDE(5-OH) and *R* was much better than that between BDE(4′-OH)/BDE(5-OH) and *F*. However, a contrasting result occurred for BDE(7-OH). Therefore, the electronic effect of the substituent at the C3 position on BDE(4′-OH) and BDE(5-OH) is mainly governed by the resonance effect, while that on BDE(7-OH) is mainly controlled by the field/inductive effect.

#### 2.3.2. Ionization Potential

The calculated IPs for the investigated compounds are given in Table 2. It is well known that the cation radical is more stable and the conjugation of the π-electrons is more delocalized in polar media [21,22]. Therefore, as can be seen in Table 2, the IPs dramatically decreased with the increasing polarity of the studied environments. Additionally, the IP of the NH_2_-substituted derivative was smaller than that of the other compounds. Thus, in the SET-PT mechanism, the NH_2_-substituted derivative was the strongest antioxidant among the studied compounds.

To study the substituted effects on the IP, the Pearson correlation was conducted. As can be seen in Figure 2, the *P* (IP, *σ*) was positive and larger than 0.8 in the studied phases, indicating that the IP was positively and highly relevant with the Hammett sigma constant. The positive coefficient suggested that the electron-withdrawing groups raised the IP and the electron-donating groups had the opposite effect. Therefore, the electron-donating groups at C3 position delocalized the π-electrons of the cation radicals, which could enhance the antioxidative activity of apigenin, while the electron-withdrawing groups at the C3 position played the opposite role in the SET-PT mechanism.

As can be seen in Figure 2, the *P* (IP, *σ*_p_) was larger than *P* (IP, *σ*_m_) and was approximate to 1. Therefore, the correlation between the IP and *σ*_p_ was better than that between the IP and *σ*_m_. *σ*_p_ could be used to predict the IP of the C3 position substituted apigenin and the linear formulas related with the *σ*_p_ and the IP are listed in Table S1 (in Supplementary Materials), which can be used to predict the IP by the *σ*_p_. In Figure 2, it also showed that the correlation between IP and *R* was much better than that between IP and *F*. Therefore, the electronic effect of the substituent on the IP was mainly governed by the resonance effect.

#### 2.3.3. Proton Affinity

The calculated PAs of different hydroxyl groups in the investigated compounds are listed in Table 3. In the gas and benzene phases, it was clearly observed that the PA of 4′-OH was smaller than that of the other hydroxyl groups. In the water phase, the PA of 7-OH was the smallest. Therefore, for the investigated compounds, the proton-donating ability of the 4′-OH group was larger than the other hydroxyl group in the gas and benzene phases, while that of the 7-OH group was the highest in the water phase. Besides, the strongest antioxidative group of the apigenin derivative was the same as apigenin in different phases. The lowest PA of the NO_2_-substituted derivative was smaller than that of the other compounds. Thus, in the SPLET mechanism, the NO_2_-substituted derivative was the strongest antioxidant among the studied compounds.

The Pearson correlation coefficients between the Hammett sigma constants, the field/inductive parameter and resonance parameter with the PAs are drawn as histograms in Figure 2. As can be seen in Figure 2, all of the *P* (PA, *σ*) were negative. Besides, the absolute values of the *P* (PA, *σ*) were all larger than 0.8, indicating that the PAs were negatively and highly relevant with the Hammett sigma constants. The negative coefficient also suggested that the electron-withdrawing groups reduced the O-H PA and the electron-donating groups had the opposite effect. Therefore, the electron-withdrawing groups at the C3 position strengthened the antioxidative activity of apigenin while the electron-donating groups had the opposite effect in the SPLET mechanism.

For 4′-OH, the absolute value of *P* (PA, *σ*_p_) was larger than that of *P* (PA, *σ*_m_) in the studied phases, while for 5-OH and 7-OH, the absolute value of *P* (PA, *σ*_m_) was larger than that of *P* (PA, *σ*_p_). Therefore, in the studied phases, for 4′-OH, the correlation between the PA and *σ*_p_ was better than that between the PA and *σ*_m_. For 5-OH and 7-OH, the result was to the contrary. Besides, the PA of 4′-OH in the substituted apigenin could be predicted better by *σ*_p_, while that of 5-OH and 7-OH could be forecasted well by *σ*_m_. The linear formulas related with the Hammett sigma constants and the PAs are listed in Table S1 (in Supplementary Materials), which can be used to predict the PAs by the Hammett sigma constants. When analyzing the electronic effects of the substituent, it was found that the correlations between PA(4′-OH) and *R* were much better than that between PA(4′-OH) and *F*, while contrary results occurred for the PA(5-OH) and PA(7-OH). Therefore, the electronic effect of the substituent on the PA(4′-OH) was mainly governed by the resonance effect, while that on the PA(5-OH) and PA(7-OH) was mainly controlled by the field/inductive effect.

## 3. Discussion

In general, free energy (∆_r_*G* = ∆_r_*H* − *T*∆_r_*S*) is the criterion to distinguish the thermodynamically favored process. However, in the case of the studied mechanisms, the absolute values of the entropic term (−*T*∆_r_*S*) reached only a few units of kJ/mol and the free energies only shifted in comparison to the corresponding enthalpies (∆_r_*H*).

For the multiple step mechanisms, the first step is thermodynamically significant. IP and PA are the enthalpies related to the first step of SET-PT and SPLET mechanisms. Based on the BDE, IP, and PA values, it is possible to estimate which antioxidative mechanism is thermodynamically preferable. Analyzing the data in Table 1, Table 2 and Table 3, it can be deduced that in the gas and benzene phases, the lowest BDEs were significantly smaller than the corresponding IPs and the lowest PAs for the studied flavonoids. Therefore, in the gas and benzene phases, the HAT is the most possible mechanism for the investigated compounds to undergo the free radical scavenging progress. In the water phase, the high solvation enthalpies of ionic species induced the PAs to drastically decrease and the lowest PAs of apigenin and its derivatives were significantly smaller than the corresponding lowest BDEs and the IPs. Thus, in the H_2_O phase, SPLET was more favorable than HAT and SET-PT in the free radical scavenging progress of the investigated compounds.

The antioxidative activity of flavonoids in the HAT and SPLET mechanisms is determined by the lowest BDE and lowest PA, respectively. In the gas and benzene phases, the electron-withdrawing groups at the C3 position reduced the antioxidative activity of apigenin while the electron-donating groups had the opposite effect. In the water phase, it was to the contrary. Therefore, the NH_2_-substituted derivative was the strongest antioxidant in the gas and benzene phases, while the NO_2_-substituted derivative was the strongest one in the water phase.

## 4. Materials and Methods 

### 4.1. Computational Details

The details of the HAT, SET-PT, and SPLET mechanisms are present in Equations (1)–(5) [24,25,26].

(1) R·+ ArOH→RH+ArO·

(2) $$R\cdot +\;\mathrm{ArOH}\to R^{-}+{\mathrm{ArOH}}^{+}$$

(3) $$R^{-}+{\mathrm{ArOH}}^{+}\cdot \to \mathrm{RH}+\mathrm{ArO}\cdot$$

(4) $$\mathrm{ArOH}\to {\mathrm{ArO}}^{-}+H^{+}$$

(5) $${\mathrm{ArO}}^{-}+R\cdot \to \mathrm{ArO}\cdot +\;R^{-}$$

In the HAT mechanism (Equation (1)), the flavonoid (ArOH) traps the free radical via the homolytic dissociation of the O-H bond. The numerical parameter associated with this mechanism is BDE (Equation (6)). The lower the BDE, the stronger the antioxidative activity of the flavonoid. SET-PT and SPLET are two-step mechanisms. In the SET-PT mechanism, an electron donates from the ArOH (Equation (2)), followed by a proton transferring from the formed cation radical (Equation (3)). The first step and second step are characterized by IP (Equation (7)) and PDE (Equation (8)), respectively. In the SPLET mechanism, a proton transfers from the antioxidant (Equation (4)), followed by an electron donated from the anion created in the first step (Equation (5)). The first step and second step are characterized by PA (Equation (9)) and ETE (Equation (10)), respectively. The calculated gas and solvent phases *H*(H^+^), *H*(e^−^), and *H*(H·) were obtained from the literature [34,35,36].

(6) BDE=H(ArO·)+H(H·)−H(ArOH)

(7) $$\mathrm{IP}=H(\mathrm{ArOH}{)}^{+}\cdot +H\left(e^{-}\right)-H\left(\mathrm{ArOH}\right)$$

(8) $$\mathrm{PDE}=H\left(\mathrm{ArO}\right)+H\left(H^{+}\right)-H\left({\mathrm{ArOH}}^{+}\cdot \right)$$

(9) $$\mathrm{PA}=H\left({\mathrm{ArO}}^{-}\right)+H\left(H^{+}\right)-H\left(\mathrm{ArOH}\right)$$

(10) $$\mathrm{ETE}=H\left(\mathrm{ArO}\cdot \right)+H\left(e^{-}\right)-H\left({\mathrm{ArO}}^{-}\right)$$

The electron-donating power is defined as

(11) $$\omega^{-}=\frac{{\left(3I+A\right)}^{2}}{16\left(I-A\right)}$$

whereas the electron-accepting power, may be defined as

(12) $$\omega^{+}=\frac{{\left(I+3A\right)}^{2}}{16\left(I-A\right)}$$

In Equations (11) and (12), *I* and *A* are vertical ionization energy and vertical electron affinity of the compound, respectively.

The calculated equations for electron acceptance index (*R*_a_) and electron donation index (*R*_d_) are

(13) $$R_{a}=\frac{\omega_{L}^{+}}{\omega_{F}^{+}}$$

(14) $$R_{d}=\frac{\omega_{L}^{-}}{\omega_{Na}^{-}}$$

In this work, for F and Na, experimental values of *I* and *A* were used. The *I*(F), *I*(Na), *A*(F), and *A*(Na) are 17.54 eV, 5.14 eV, 3.40 eV, and 0.54 eV, respectively. The $\omega_{F}^{+}$ and $\omega_{Na}^{-}$ calculated using the experimental values of *I* and *A* were 3.40 and 3.46, respectively.

All calculations were carried out using the DFT method implemented in the Gaussian 09 package [37]. The optimized geometries, vibrational frequencies, distributions, and energies of the frontier orbitals were obtained by using the M062X method with a 6-31G* basis set. No constraints were imposed on the geometries and all possible conformers for the radicals and neutral species were investigated. The conformer with the lowest electronic energy was confirmed by the true minima on the calculated potential surface and verified by the absence of the imaginary frequency. Single point energy calculations were performed at the M062X/6-311+G** level of theory using the optimized geometries. The solvent effect was performed using the SMD continuum solvent model.

### 4.2. Statistics Analysis

In statistics, the Pearson correlation is an effective method to study a broad class of relationships among variables [38]. It is based on the covariance matrix of the data and the Pearson correlation coefficient is the parameter to evaluate the strength of the relationship between two vectors. Normally, the Pearson correlation coefficient (*P*_x,y_) between two vectors *x* and *y* is

(15) $$P_{x,y}=\frac{\sum xy-\frac{\sum x\sum y}{N}}{\sqrt{(\sum x^{2}-\frac{\left(\sum x^{2}\right)}{N})\left(\sum y^{2}-\frac{\left(\sum y^{2}\right)}{N}\right)}}$$

where *N* refers to the size of the signature array and was 11 in this work. The Pearson correlation coefficient is symmetric: *P*_x,y_ = *P*_y,x_. The *P*_x,y_ is between 1 to −1. The *P*_x,y_ equal to 1 or −1 corresponds to data points lying exactly on a line, or to a bivariate distribution entirely supported on a line.

## 5. Conclusions

The relationship between the substituent and antioxidative activity of flavonoids has not yet been completely elucidated. In this work, the DFT methods were applied to investigate the influence of the substituent on the antioxidative activity of flavonoids based on the HAT, SET-PT, and SPLET mechanisms from the thermodynamic aspect. A series of apigenin derivatives with different substituents at the C3 position were selected. DAM analysis illustrated that the studied compounds are worse electron acceptors than F and also are not better electron donors than Na. The strongest antioxidative group of the apigenin derivative was the same as the apigenin in different phases. Excellent correlations were found between the BDE/IP/PA and Hammett sigma constants. Therefore, Hammett sigma constants can be used to predict the radical scavenging activities of the substituted apigenin and to design new antioxidants based on flavonoids. It was also found that:

(1) The electronic effect of the substituent on the BDE and PA of 4′-OH and IP was mainly governed by the resonance effect, while that on the BDE and PA of 7-OH was mainly controlled by the field/inductive effect. For 5-OH, the BDE and PA were more influenced by the resonance effect and field/inductive effect, respectively.
(2) The substituent effects on BDE and IP were very different from those on PA. The electron-withdrawing groups enhanced BDE and IP, while the electron-donating groups reduced the BDE and IP. The effect of the substituents on PA was to the contrary.
(3) In the gas and benzene phases, the free radical scavenging progress of the investigated compounds underwent the HAT mechanism with the most possibility. In the water phase, SPLET was the most favorable mechanism. Therefore, in the gas and benzene phases, the electron-withdrawing groups at the C3 position reduced the antioxidative activity of apigenin while the electron-donating groups had the opposite effect. In the water phase, it was to the contrary. Furthermore, the NH_2_-substituted derivative was the strongest antioxidant in the gas and benzene phases, while the NO_2_-substituted derivative was the strongest antioxidant in the water phase.

## Figures and Tables

**Table 1:** O-H bond dissociation enthalpy (BDE) in kJ/mol obtained by the M062X/6-311+G** method. The data in bold and underline represent the lowest BDEs.

	Gas			Benzene			Water		
	4′-OH	5-OH	7-OH	4′-OH	5-OH	7-OH	4′-OH	5-OH	7-OH
H	**368.6**	435.2	387.4	**372.2**	429.6	391.6	**368.1**	393.7	390.2
NH_2_	**357.4**	424.7	384.4	**359.4**	419.3	386.5	**351.0**	383.6	379.1
OMe	**366.2**	430.7	387.3	**370.0**	425.9	391.2	**367.4**	391.9	389.2
Me	**369.2**	432.8	387.1	**372.7**	427.5	391.0	**368.8**	391.6	388.7
OH	**360.5**	422.8	386.9	**363.7**	417.6	390.2	**357.9**	384.9	385.5
F	**369.8**	431.2	389.4	**373.9**	425.2	393.8	**370.6**	390.6	392.1
Cl	**372.3**	432.6	389.9	**376.6**	426.8	394.7	**373.8**	392.4	393.4
CHO	**374.0**	435.5	391.2	**377.1**	430.2	395.8	**373.1**	396.5	395.0
CF_3_	**376.2**	434.3	391.4	**380.4**	428.5	396.4	**377.0**	394.7	395.8
CN	**378.5**	435.9	392.2	**382.9**	429.8	397.4	**379.3**	395.9	397.1
NO_2_	**378.4**	433.8	411.0	**382.7**	427.6	416.8	**380.0**	395.5	416.4

**Table 2:** Ionization potential (IP) in kJ/mol obtained by the M062X/6-311+G** method.

	Gas	Benzene	Water
H	792.9	684.0	589.1
NH_2_	703.0	591.8	503.6
OMe	735.2	633.4	556.9
Me	779.8	671.8	586.2
OH	740.8	636.3	548.8
F	790.1	680.4	590.4
Cl	793.4	689.1	603.0
CHO	814.3	702.1	606.1
CF_3_	815.0	703.2	604.6
CN	827.3	718.5	631.5
NO_2_	833.0	724.8	636.8

**Table 3:** Proton affinity (PA) in kJ/mol obtained by the M062X/6-311+G** method. The data in bold and underline represent the lowest PAs.

	Gas			Benzene			Water		
	4′-OH	5-OH	7-OH	4′-OH	5-OH	7-OH	4′-OH	5-OH	7-OH
H	**1354.8**	1453.7	1378.4	402.0	476.4	411.7	146.9	162.5	**139.1**
NH_2_	**1371.2**	1446.1	1377.8	412.6	471.2	414.7	155.4	164.9	**144.2**
OMe	**1364.0**	1445.2	1374.0	407.6	470.1	408.9	148.0	162.2	**140.2**
Me	**1366.5**	1456.4	1382.3	411.4	478.3	414.3	154.8	165.3	**141.5**
OH	**1362.0**	1423.5	1368.0	400.4	452.0	410.6	152.2	156.2	**140.0**
F	**1353.0**	1434.8	1364.7	399.1	459.6	400.8	147.0	155.9	**136.8**
Cl	**1355.3**	1436.1	1364.8	400.5	460.7	402.0	151.2	156.6	**135.6**
CHO	**1340.1**	1426.6	1353.9	389.4	456.1	394.1	141.2	155.7	**133.9**
CF_3_	**1341.9**	1426.5	1355.4	391.5	453.5	393.7	146.8	153.2	**133.1**
CN	**1329.2**	1417.5	1345.7	381.5	447.2	387.3	141.4	151.9	**131.8**
NO_2_	**1330.4**	1411.7	1343.0	382.3	442.7	385.7	137.8	149.0	**130.0**

## Supplementary Materials

The following are available online, Figure S1: The energies and distributions of HOMO and LUMO orbitals for the investigated compounds in the gas phase; Figure S2: DAM for apigenin and its derivatives. The inset is definition of the four regions in DAM; Table S1: The linear formulas related with the Hammett sigma constants and the BDE/IP/PA. In the formulas, y represents the BDE/IP/PA, x represents the Hammett sigma constants.

## Abbreviations

DFT	density functional theory
HAT	hydrogen atom transfer
SPLET	sequential proton loss electron transfer
SET-PT	single electron transfer followed by proton transfer
ArOH	flavonoid
BDE	bond dissociation enthalpy
IP	ionization potential
PDE	proton dissociation enthalpy
PA	proton affinity
ETE	electron transfer enthalpy
Δ_r_*G*	Gibbs free energy
*σ*	Hammett sigma constants
*F*	field/inductive effect
*R*	resonance effect
