# Supplementary material for: The Substituent Effect on the Radical Scavenging Activity of Apigenin
Source: Molecules. 2018 Aug 10;23(8):1989. doi: 10.3390/molecules23081989 (PMC6222755; doi:10.3390/molecules23081989)
Supplement: Supplementary file 1 [file molecules-23-01989-s001.pdf]

Supplementary materials for:

The substituent effect on the radical scavenging activity of apigenin

Yan-Zhen Zheng[1,‡], Da-Fu Chen[1,*,‡], Geng Deng[2] and Rui Guo[1]

1 College of Bee Science, Fujian Agriculture and Forestry University, Fuzhou 350002, P. R. China; yanzhenzheng@fafu.edu.cn (Y.Z.Z.); dfchen826@fafu.edu.cn (D.F.C.); rui_0508@163.com (R.G.)

2 Key Laboratory of Bioorganic Phosphorous Chemistry and Chemical Biology (Ministry of Education), Department of Chemistry, Tsinghua University, Beijing 100084, P. R. China; dengg13@mails.tsinghua.edu.cn (G.D.)

‡ These authors contributed equally to this work

* Correspondence: dfchen826@fafu.edu.cn (D.F.C.); Tel.: +86-0591-8378-9482

Table S1. The linear formulas related with the Hammett sigma constants and the BDE/IP/PA. In the formulas, y represents the BDE/IP/PA, x represents the Hammett sigma constants

	gas	benzene	water
BDE(4′−OH) vs σ_p	y = 14.5x + 368.5	y = 15.7x + 372.0	y = 18.1x + 367.7
BDE(5−OH) vs σ_p	y = 7.4x + 431.0	y = 6.7x + 425.4	y = 8.1x + 391.0
BDE(7−OH) vs σ_m	y = 9.9x + 386.7	y = 13.2x + 390.1	y = 19.9x + 386.5
IP vs σ_p	y = 86.4x + 774.5	y = 84.3x + 666.6	y = 79.3x + 578.2
PA(4′−OH) vs σ_p	y = -30.0x + 1355.0	y = -21.4x + 400.4	y = -10.5x + 148.7
PA(5−OH) vs σ_m	y = -46.4x + 1446.1	y = -38.2x + 470.3	y = -19.0x + 162.4
PA(7−OH) vs σ_m	y = -46.9x + 1376.2	y = -37.9x + 411.7	y = -15.9x + 140.9

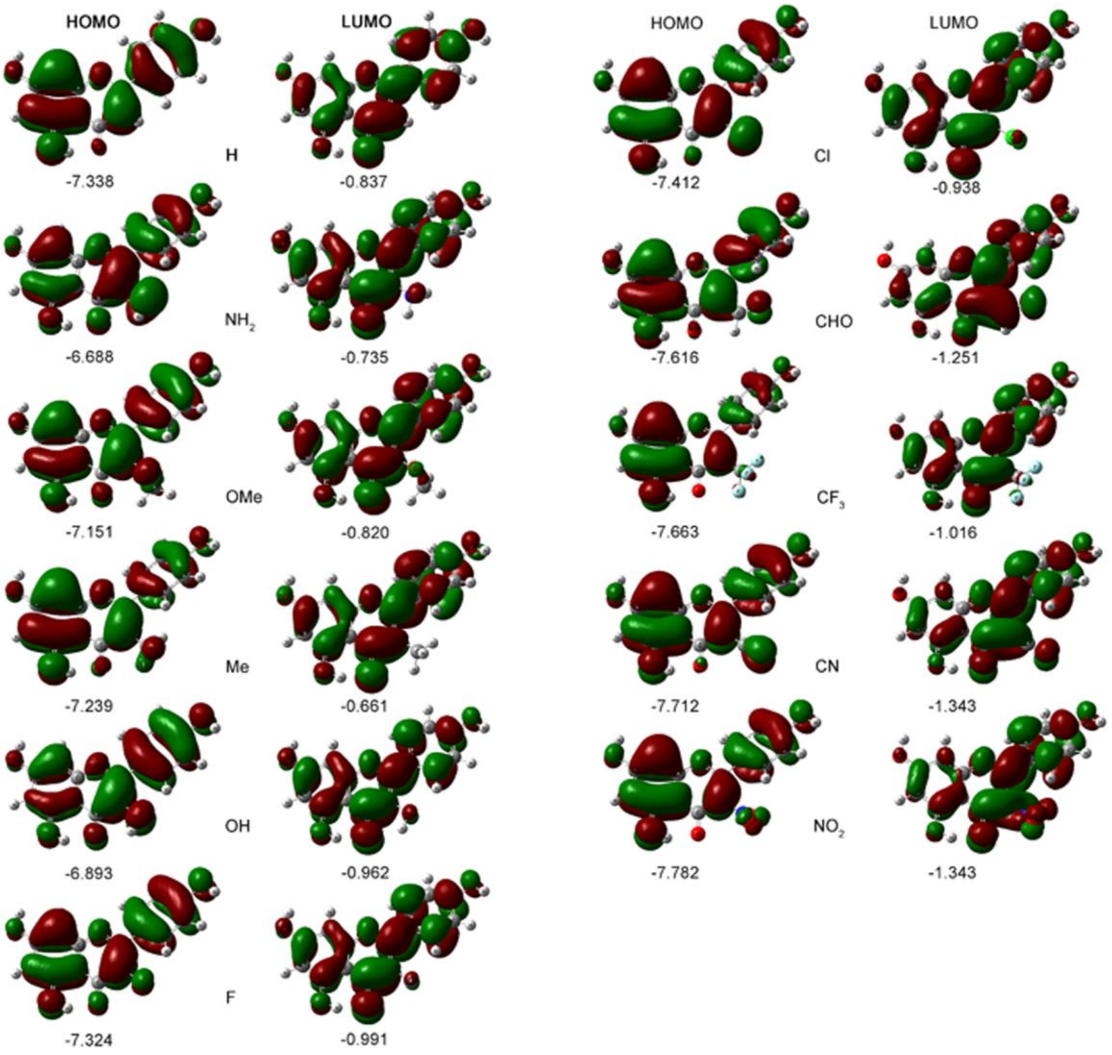

Figure S1. The energies and distributions of HOMO and LUMO orbitals for the investigated compounds in the gas phase.

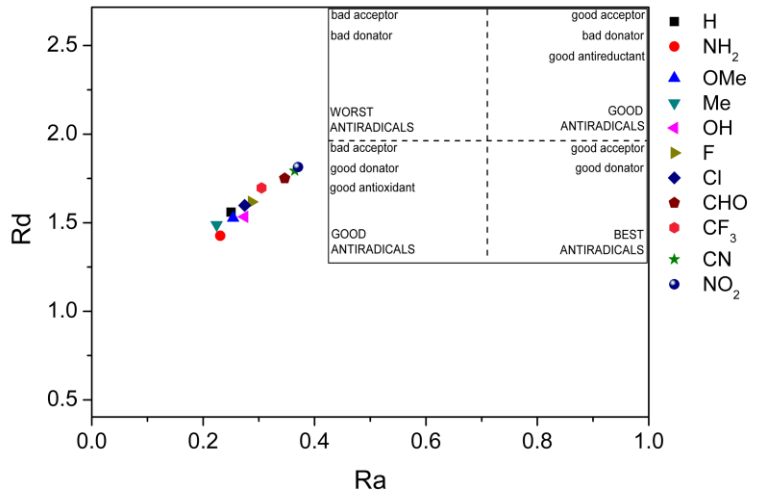

Figure S2. DAM for apigenin and its derivatives. The inset is definition of the four regions in DAM.
